# Career flexibility and its relation to time perspective: a study with college students in the Portuguese context

**DOI:** 10.3389/fpsyg.2023.1078752

**Published:** 2023-05-22

**Authors:** Juliana Frainer, Isabel N. Janeiro

**Affiliations:** CICPSI, Faculdade de Psicologia, Universidade de Lisboa, Alameda da Universidade, Lisbon, Portugal

**Keywords:** career flexibility, time perspective, planned happenstance theory, higher education students, confirmatory factor analysis

## Abstract

The volatility of the labor market resulting from globalization, rapid technology changes, economic competition, and the impact of events such as the Covid-2019 Pandemic, demand from vocational psychology a more refined understanding of the processes individuals face while dealing with those new challenges and opportunities, especially in uncertain contexts. Theories such as Planned Happenstance address constructs such as career flexibility, an essential skill to recognize, create and use chance events as career opportunities. Furthermore, when fortuitous events and uncertain contexts are considered for career development, it becomes relevant to understand how subjective time perspective evolves, that is how the life events and career goals are projected, accessed, valued, and organized. Given this context, the objectives of the present study are to adapt and validate a Portuguese version of the Career Flexibility Inventory and to explore the possible relationships between career flexibility, time perspective, and variables inherent to the educational context. The Portuguese version of the Career Flexibility Inventory, the Time Perspective Inventory and a sociodemographic form were answered by 1,380 students from Portuguese higher education institutions. The results indicated that the Portuguese version of the CFI has an adequate three-factor structure with good reliability indices. Some limitations regarding psychometric validity show the importance of further research to improve the measure. However, the findings contribute to theoretically and operationally deepening discussions on the multidimensionality of Career Flexibility. The results regarding the relationships between time perspective and career flexibility seem to be in line with the theoretical indicators of the variables and support the formulated hypotheses, specifically, that future orientation presents a positive correlation with active adaptation; a negative correlation with wavering, and wavering presents a positive correlation with the negative future orientation. The results partially support the hypothesis of differences on time perspective and career flexibility among students with different academic grade averages and from diverse scientific areas of study. Finally, the study advances a theoretical reflection on the different nature of career flexibility dimensions and contributes to broadening and promoting theoretical and operational discussions on the relationships between time perspective and career flexibility, which are still incipient.

## Introduction

Career development involves complex and dynamic processes with intertwined movements between various aspects and contexts of life. It includes competencies needed to deal with the labor market but also personal beliefs and attitudes that shape the different individual career paths (Savickas, 2020). Due to factors such as globalization, rapid technological changes, and economic competition, skills that used to be highly valued have become obsolete as the opportunities and requirements of different professions change, new jobs are created and others are abandoned (e.g., Allen and Velden, 2012; Karaca-Atik et al., 2023). Even in the so-called essential jobs, a reduction in identifiable and predictable career paths is evident (Savickas, 2011).

In addition, the recent events of the Covid-2019 Pandemic have generated even more economic hardship, social transformations, and evidenced contexts immersed in uncertainty (Venkatachary et al., 2020). New ways of organization of work practices and education, especially with the use of technologies and online activities (García-Peñalvo et al., 2021) emerged at a rapid pace. In this sense, there is a growing need for career psychology to better understand the processes by which individuals seek different career paths as they continuously face new challenges and opportunities (Lee et al., 2011), especially in these contexts of uncertainty (Krumboltz, 2009). To capture the ongoing social and economic transformations, traditional career theories (e.g., Holland, 1959; Super, 1995), are being replaced or adapted, and concepts such as adaptability (Savickas, 2011), flexibility, or chance (e.g., Mitchell et al., 1999; Krumboltz, 2009) are considered fundamental attributes to deal with the new career challenges.

Recently, Kim (2019) developed a new inventory for measuring career flexibility called Career Flexibility Inventory (CFI). Through the scope of the Planned Happenstance Theory (PHT), career flexibility is characterized by the ability of individuals to adapt or adjust their attitudes, beliefs, and career trajectories to events that are inevitable in the contexts into which they are inserted. Furthermore, this skill is considered essential to enable individuals to recognize, create and effectively utilize these opportunities for career development (Mitchell et al., 1999).

One of the main goals of career development literature is to understand how individuals design, access, value and organize their life events (Sampson et al., 2022) and establish and prioritize their career goals. For example, studies in the European context indicated that the pandemic situation negatively and expressively affected “on young people’s mental well-being, as a result not only of loss of jobs and educational opportunities but also of restrictive measures resulting in reduced social contact and delayed of the future plans.” (Eurofound, 2021, p. 75). Thus, time perspective is evidenced as a primordial construct for career development (Janeiro, 2010; Park et al., 2021) because the perception of time relative to previous experiences, the moment of choice and the moment of reaching an objective, linked to fortuitous events, can significantly influence, or impact the career trajectory (Hesketh, 2000).

Although some studies explore the effects of variables in the university context with different career-related constructs (e.g., Abe et al., 2021; Bennett et al., 2021; Kim et al., 2023), there is a lack of studies that address the possible relationships between career flexibility, time perspective, and educational variables, such as the scientific area of study or academic grades.

Given the above background, and with the proposal to expand theoretical and practical contributions, namely on the cognitive, affective and motivational dimensions linked to career development, the objectives of this study are to adapt the CFI (Kim, 2019) for use with **college students** in Portugal; to explore the possible relationships between career flexibility and time perspective; and additionally to explore differences in career flexibility and time perspective between groups of students when considering some academic variables.

### Career flexibility

The main idea of the PHT is to explore how casual events can inevitably play an important role in the development of career trajectories, and how events attributed to chance can often be indirect results of effective behavior (Mitchell et al., 1999). This theory identifies five skills that facilitate the recognition, creation, and maximization of the use of chance as a career opportunity: (a) curiosity: the ability to explore new learning opportunities, (b) persistence: the ability to continue their efforts in the face of challenges, despite setbacks, (c) flexibility: the ability to adapt, change or adjust their attitudes and circumstances in the face of uncertain contexts, (d) optimism: the ability to identify new opportunities and perceive them as possible and attainable, and (e) risk-taking: the ability to act in the face of uncertain results.

Considered one of the core skills identified by the PHT, career flexibility can be defined generically as the degree to which a decision leading to action allows the individual to have other choices over time (Yahanpath et al., 2013). According to this theory, one needs to be aware that unplanned events tend to occur, and having a flexible attitude makes it possible to be prepared to find, assimilate, and grasp those opportunities or events attributed to chance (Mitchell et al., 1999). Flexibility is essential, for example, to assess the risks associated with a particular course or career choice (Yahanpath et al., 2013). Together with optimism, it predicts psychological well-being and can be considered a significant individual strength that contributes to the sustainable development of each person in different contexts (Valickas et al., 2019).

August-Brady (2000) examined the concept of flexibility and identifies it as a complex and multidimensional construct defined as an evolutionary and resilient response to recognized changes and uncertainties based on openness and willingness to change. Flexibility allows attention to be directed towards a greater diversity of choices and greater effectiveness and efficiency in results; in this way, exploration tends to be a notable attribute of individuals with flexible attitudes (Kim et al., 2023).

Career flexibility can be characterized as having an active nature, that is, the ability to respond and adapt to different purposes, conditions, or changes when performing actions effectively and efficiently is evidenced. It is noteworthy that inherent to this active nature is the ability to change or respond to changes without external intervention. In other words, resilience is an attribute that seems to denote the active nature of flexibility (August-Brady, 2000).

However, Kim (2019) argues that, in the definition of career flexibility, only the active nature of the construct is considered (e.g., Mitchell et al., 1999; Krumboltz, 2009), when it would be ideal to add to the concept, a passive nature too. The passive nature of flexibility is characterized as a state that is easily changed by external factors and moves with pressure, that is, passively flexible individuals do not commit to actions toward a goal (August-Brady, 2000; Kim, 2019). The passive nature of flexibility encompasses states of stagnation and indecision, which, in turn, can play a dysfunctional role in career development and cause anxiety and psychological distress (Gati, 2013; Kim et al., 2020).

To assess career flexibility more accurately and considering its multidimensionality, that is, the passive nature and the active nature of the construct, Kim (2019) developed a new instrument, the Career Flexibility Inventory (CFI) and identified three main dimensions of career flexibility, namely: (a) wavering: represents the passive aspect of flexibility. Passively flexible individuals do not present attitudes and behaviors that prioritize their goals, as the behavior is moving by external stimuli and pressures, and not necessarily so that a goal is effectively achieved. Passively flexible people sometimes feel indecisive about career choices and goals, (b) active adaptation: represents the proactive aspect of flexibility and is characterized by active behavior in the face of opportunities, marked by a proactive approach to changes and that considers productive alternatives with certain long-term career goals, and (c) flexible thinking: characterized by the ability to accept unplanned changes and to present an open attitude toward possible instabilities in career trajectories. This factor represents the reactive aspect of career flexibility. In summary, wavering refers to the passive nature of flexibility, and active adaptation and flexible thinking represent the active nature of flexibility.

### Time perspective and academic variables

As a multidimensional construct, time perspective can be defined as the process by which the continuous flow of existence is grouped into three temporal categories, the past, the present, and the future. These categories help individuals to attribute order, coherence, and meaning to life events (Nuttin and Lens, 1985; Zimbardo and Boyd, 2008). Effectively, developing a balanced time perspective means having an awareness of what the past and present are and how to project themselves into the future, and this awareness is essential for individuals to be able to plan their goals and to make career decisions (Kirdök, 2018).

Individuals often exhibit a dominant time orientation, linked to various behavioral and psychological outcomes. For instance, individuals with a dominant future orientation focus predominantly on future goals and tend to be self-disciplined and perseverant. People with an orientation to the past tend to value traditions and have a posture of resistance to social changes, whereas individuals with a present orientation tend to be more impulsive and extroverted and are willing to focus on enjoying the moment (Zimbardo and Boyd, 2008).

Future orientation appears to be essential for motivation, engagement, and formation of career-related interests (Taber, 2013; Imbellone and Laghi, 2016), as well as for career maturity (Janeiro, 2010) and career decision-making self-efficacy (Park et al., 2020; Kvasková and Almenara, 2021). Low levels of future time perspective are associated with career choice anxiety (Park et al., 2018) and career indecision (Ferrari et al., 2010). Present hedonistic, a negative vision of the future and past negative orientation, all seem to contribute to various career decision difficulties (Taber, 2013) and, when taken together, also contribute to low career decision-making self-efficacy (Kvasková and Almenara, 2021).

In the educational context, academic performance and the scientific area of study have been objects of increasing attention in career psychology due to the remarkable impacts it can have on career decisions, course satisfaction, and future plans (e.g., Cox et al., 2016; Maksimovic et al., 2020; Bennett et al., 2021; Boo et al., 2022). When related to time perspective, studies indicate, for example, that future orientation has a positive relationship with higher academic levels (Ferrari et al., 2010) and students with this orientation tend to have a more goal-oriented behavior, which can result in better grades in the long run (Barnett et al., 2020). Present orientation, on the other hand, appears to be negatively associated with academic engagement. Regarding the scientific areas of study, Bennett et al. (2021) identified that medical and health students were more confident in their career decisions than colleagues who attended courses in other scientific areas. However, these same students were less aware of alternative career paths and less prepared to reorient their careers if necessary.

### Objectives and hypothesis

The main objective of this study is to adapt the CFI (Kim, 2019) to the Portuguese context. Additionally, we intend to explore the relationships between career flexibility and time perspective and to investigate whether these factors differ between student groups based on their academic grades and scientific area of study.

Given the multidimensionality of both constructs, we expect that their relationship will vary in different ways. Specifically, as active adaptation and flexible thinking represent the active nature of flexibility and future orientation refers to a positive view of the future with a focus predominantly on future goals, it is expected that:

*H1*: active adaptation and flexible thinking will be positively correlated with future orientation. On the other hand, wavering captures the passive nature of career flexibility, and present oriented individuals live for the moment and are less engaged in their career plans, so it is expected that:

*H2*: wavering will be positively correlated with present orientation and negatively correlated with future orientation. Furthermore, based on empirical evidence about the relationships between time perspective, academic achievement, and scientific areas, the following hypotheses are formulated:

*H3*: students who demonstrate future orientation, active adaptation, and flexible thinking will have higher grade levels compared to those who have negative future orientation and wavering.

*H4*: there will be differences in time perspective and career flexibility between students studying in different scientific areas of study.

## Method

### Participants

The minimum sample size of this study was estimated based on the recommendations by Hair et al. (2018), who consider that for a significance level of 5%, and power of 80% it is necessary to include 5 to 20 observations per study variable. The requirements regarding the minimum sample size for the multivariate analysis of variance (MANOVA) (Hair et al., 2018) were also considered. Therefore, for this study, *a priori* minimum of 300 participants was estimated. Initially, 1.420 participants, randomly selected from different Portuguese higher education institutions, responded to the survey. However, 40 observations (2,82%) were considered null, due to non-responses. From the total sample of 1.380 participants, 423 are male students (31%) and 957 female students (69%), and the ages ranged between 18 and 34 years (M = 22.12; SD = 3.6).

The research sample included students from 262 different courses (bachelor’s, master’s, integrated master’s, doctoral, and other postgraduate courses). For data analysis, the courses were organized into seven scientific areas (based on FCT – Foundation for Science and Technology classification), so the sample comprises 44% students from Social Sciences, 21% of Engineering Sciences and Technology, 12% of Humanities, 11% of Medicine and Health Sciences, 5% of Agricultural Sciences, 4% of Natural Sciences, and 2% of Exact Sciences.

The academic grades were organized into five levels in accordance with the European rating comparability scale, namely: E – values between 10–11 (2.4% of students), D – values between 12–13, (22% of students), C – values between 14–15 (41.5% of students), B – values between 16–17, (23.8% of students), and A – values between 18–20, (4.3% of students).

### Procedures

The study was approved by the Deontology Committee of the School of Psychology of the University of Lisbon.

Some sample inclusion criteria were defined, namely: (a) higher education students and (b) students aged 18 to 34 years old, age group characterized as young adults (Levinson, 1986; Arnett, 2000). For sample selection, emails were sent to course coordinators asking them to invite their students to participate in the survey. This e-mail contained all the information related to the objectives of this study and the instructions for accessing the survey.

The instruments were presented online [Qualtrics Surveys platform (version 1.2020), 2005] and the link to the survey was active from January to March 2022; the response time for each participant was 15 min on average.

Participation was voluntary and was conditioned on acceptance of the Informed Consent Form, made available in an online format. Data collection respected all ethical requirements and met the criteria for participant anonymity.

### Measures

(a) Career Flexibility Inventory (CFI; Kim, 2019) is an inventory organized by Kim (2019) to assess career flexibility. It consists of a total of 15 items distributed in three dimensions, each one with five items, namely: (a) wavering, comprising items such as “I tend to adjust my career to given circumstances without having any long-term outlooks”, (b) active adaptation, “When it comes to choosing my career, I try to find various alternatives”, and (c) flexible thinking, “It is okay for me to change my career.” Two reverse-worded items have been included in the inventory (items 14 and 15) to inhibit random responses. In the inventory, the respondent is asked to evaluate how well each item describes him or her on a 5-point Likert scale, from 1 (*never true*) to 5 (*always true*). The first validity studies of the inventory in a sample of South Korean college students indicated adequate psychometric properties, and the model hypothesized of three dimensions was also acceptable (CFI = 0.91; TLI = 0.90; RMSEA = 0.07) (Kim, 2019).

The translation of the English version of the inventory involved an evaluation panel composed of researchers with proficiency in English and Portuguese languages, knowledge of vocational psychology, and familiarity with the cultural context of this study. A translation from English into Portuguese was prepared with subsequent back-translation of the instructions and items (semantic and lexical meanings) (Hambleton, 2005). No significant discrepancies were found between the versions designed by the specialists, and, after the final analysis of the inventory, a definitive version of the CFI in Portuguese was approved. A pilot study was applied to five college students in Portugal. The participants were encouraged to expose their perceptions about the content expressed in each item and, in general, no relevant doubts or difficulties were indicated.

(b) Time Perspective Inventory (Short Form) (TPI-Short Form; Janeiro et al., 2017) is an instrument organized by Janeiro (2012) to assess time perspective. The short form of TPI (Janeiro et al., 2017), has 19 items distributed into four scales (a) Future orientation scale includes 6 items that assess positive attitudes toward the future (e.g., “I have lots of projects for my future”), (b) Present orientation scale has 6 items and assesses attitudes and beliefs about the present (e.g., “I think life should be lived day by day”), (c) Past orientation scale, with 3 items, assesses attitudes toward the past (e.g., “I would like to be a child again because everything was easier then”), and (d) Negative vision of the future scale (4 items) assesses negative or anxiety-laden perceptions about the future (e.g., “I am going into the future not by choice but because I cannot stop”). Responses are indicated on a seven-point Likert scale (1. *Does not correspond at all to the way I think,* to 7. *Corresponds very strongly*). A study with the Short Form TPI conducted with a sample of 11th-grade students indicated adequate psychometric properties. The four-factor solution showed a good fit, *χ*2(144) = 334,287, *p* < 0.001, *χ*2/d*f* = 2.32, RMSEA = 0.06, CFI = 0.92, TLI = 0.91, SRMR = 0.07, with standardized factor loadings ranging from 0.46 to 0.90 (Janeiro et al., 2017).

TPI-Short Form reliability indices (*α* and *ω*) in this study’s sample were adequate for three scales, future orientation, present orientation, and negative future orientation. The past orientation scale obtained a less satisfactory coefficient (Table 1), very similar to those found in previous studies with secondary school students (Janeiro et al., 2017).

**Table 1:** Descriptive statistics, correlations, and reliability coefficients of the dimensions of career flexibility and time perspective.

Variable	M	SD	1	2	3	4	5	6	7	*α*	*ω*
1. Future	24.67	8.16	–							0.87	0.87
2. Present	23.08	7.18	−0.30^**^	–						0.79	0.79
3. Past	12.80	4.1	−0.03	0.15^**^	–					0.49	0.62
4. Negative	11.32	5.88	−0.54^**^	0.31^**^	0.13^**^	–				0.81	0.82
5. Wavering	12.19	4.00	−0.49^**^	0.26^**^	0.13^**^	0.52^**^	–			0.78	0.78
6. Flexible thinking	25.67	4.58	−0.05	0.09^**^	−0.02	−0.12^**^	0.09^**^	–		0.76	0.76
7. Active adaptation	9.35	2.17	0.33^**^	0.02	−0.06^*^	−0.26^**^	−0.15^**^	0.35^**^	–	0.60	0.61

*N* = 1.380. M, mean; SD, standard deviation; *α*, Cronbach’s alpha; *ω*, omega. *p < 0.05. **p < 0.01.

(c) Participants also completed a sociodemographic form to characterize the sample in terms of age, gender, course they were attending, and academic grades of the last year.

### Statistical analysis of the data

The normality of the data was verified using a criterion based on a sample larger than 300 participants (Kim, 2013), considering absolute values of skewness < 2 and kurtosis < 7 (West et al., 1996).

Due to the lack of empirical evidence on how the items of the CFI would aggregate in Portuguese samples, a two-step procedure was conducted. The first step involved exploring the aggregation of the items using an exploratory factor analysis (EFA) (Brown, 2006) with the principal axis factorization method and oblique Promax rotation. To analyze the data, the pattern matrix, and the structural matrix were considered (Field, 2013). The criterion for determining significant factor loadings was based on sample size. Specifically, and following Hair et al. (2018) guidelines for samples with at least 350 participants, factor loadings of 0.3 or greater were considered significant.

The criterion based on classical parallel analysis (Horn, 1965; Baglin, 2014) was used to define the number of factors to retain, and for that, the PC software package FACTOR was used (Lorenzo-Seva and Ferrando, 2021).

The second step aimed to confirm the aggregation structure of the items using confirmatory factor analysis (CFA). The statistics and quality-of-adjustment indices adopted were the values of the chi-square difference test on the Satorra-Bentler Scale, estimated by maximum likelihood (ML, a suitable estimator for data that violate the assumption of normality). In addition to these, criteria such as comparative fit index (CFI), goodness-of-fit index (GFI), root mean square error of approximation (RMSEA), and the Akaike information criterion (AIC) were considered (Marôco, 2021b). Additionally, the convergent validity was calculated using the average variance extracted (AVE) and Composite reliability (CR) (Hair et al., 2018).

It is noteworthy that the total sample of this study (*N* = 1.380) was randomly divided into groups comprising approximately 50% of the sample, with 713 participants included in the exploratory factor analysis and 676 participants in the confirmatory factor analysis.

The internal consistency reliability of the CFI was evaluated using Cronbach’s coefficient and McDonald’s Omega (McDonald, 1999; Cohen et al., 2014).

To explore the possible relationships between CFI and TPI, the results of a bivariate correlation with Pearson’s coefficient were analyzed (Callegari-Jacques, 2009; Field, 2013).

In addition, a Multivariate Analysis of Variance (One-way MANOVA) was performed to explore and evaluate possible differences in the factors ‘academic grades’ and ‘scientific areas of study’ on career flexibility and time perspective. As the assumption of multivariate normality tested by Mardia’s Test was not supported in this sample, the measure to test the statistical significance of group differences in the MANOVA was the Pillai’s criterion (Hair et al., 2018). The assumption of variance–covariance homogeneity in each group was evaluated with the Box M test, specifically: Bos M Test (*M* = 138,341; *F* (112, 65503.83) = 1,191; *p* = 0.083) for the ‘academic grades’ factor and Bos M Test (*M* = 194,886; *F* (168, 94970.52) = 1,116; *p* = 0.145) for the ‘scientific area of study’ factor.

When significant effects were detected by MANOVA, an ANOVA for each of the dependent variables was performed, followed by Tukey’s Post-hoc HSD test. The level of significance considered was *α* = 0.05 (Marôco, 2021a).

Statistical treatment of the data was performed using SPSS Statistic for Windows, v. 28 and SPSS Amos Statistic for Windows, v. 27 (IBM Corp., 2022).

## Results

### Validity and reliability of the CFI in the Portuguese context

Absolute values of asymmetry and kurtosis indicated the reasonable normal distribution of data. Using the Mahalanobis distance, ten observations were considered bivariate outliers. The mean, standard deviation, and range of the data were calculated with and without outliers and as no significant impacts were observed on the results the outliers were not removed (Manly and Navarro Alberto, 2017).

KMO results (0.80) and Bartlett test (*p* < 0.000) indicate that the EFA is adequate for the sample of this study. To determine the number of factors to retain it was used the parallel analysis procedure. This analysis indicated 3 factors to retain, explaining about 52.31% of the total variance.

Table 2 shows the results of the two matrices after oblique rotation and the factorial loadings of the CFI items.

**Table 2:** Matrices with the factor loadings of the CFI items.

CFI Items	Pattern matrix			Structure matrix		
	Factor loadings			Factor loadings		
	**1**	**2**	**3**	**1**	**2**	**3**
1	**0.58**	0.11	0.23	**0.58**	0.28	0.24
2	**0.73**	−0.09	0.04	**0.72**	0.02	−0.04
3	**0.77**	−0.05	−0.08	**0.77**	0.02	−0.14
4	**0.59**	0.06	−0.30	**0.61**	0.00	−0.30
5	**0.57**	0.05	−0.03	**0.58**	0.11	−0.04
6	0.04	−0.01	**0.62**	0.01	0.27	**0.61**
7	−0.22	−0.07	**0.71**	−0.26	0.21	**0.69**
8	0.00	0.21	**0.37**	0.01	0.37	**0.46**
9	0.14	**0.44**	0.15	0.19	**0.53**	0.34
10	0.10	**0.32**	0.15	0.13	**0.40**	0.28
11	0.09	**0.48**	0.28	0.14	**0.61**	0.49
12	−0.01	**0.68**	0.06	0.07	**0.71**	0.36
13	0.12	**0.65**	−0.01	0.20	**0.66**	0.27
14	−0.09	**0.71**	−0.20	0.01	**0.61**	0.12
15	−0.34	**0.58**	−0.09	−0.26	**0.50**	0.18
Eigenvalues	3.53	2.93	1.39	–	–	–
% total variance	23.54	19.52	9.26	–	–	–

*N* = 713. In bold, values with factorial loadings greater than 0.3. The factor loadings have standardized estimates (correlation metric).

The first factor had factor loadings greater than 0.5 in both matrices; it comprises the five items of the wavering dimension in the original inventory and explains 23.54% of the total variance. The second factor explains 19.52% of the variance and grouped seven items with factor loadings above 0.4, and one item with a loading value of 0.32. The highest factor loadings were assigned to five items that belonged to the flexible thinking dimension in the original inventory and two items from the active adaptation dimension. The third factor, which explains 9.26% of the total variance of the data, included only three items with loadings with values mostly greater than 0.6, which were part of the active adaptation dimension in the original version of the inventory.

Through CFA, we sought to evaluate the quality of fit of two hypothetical theoretical measurement models to the correlational structure of the CFI items. The first hypothetical model, called “H1:15-3 original version,” considered the structure of the CFI in terms of the grouping of the items in the original version. The second hypothetical model, called “H2:15-3PT,” assumed the results of the exploratory factor analysis performed with the Portuguese sample (Table 2). Both hypothetical models were restricted to first-order factors. Table 3 presents the summary of the goodness-of-fit indices of the hypothesized models.

**Table 3:** Adjustment indices for the confirmatory factor analyses of the CFI for the Portuguese sample.

Model	*χ*2	d*f*	CFI	RMSEA	GFI	AIC
H1:15-3 original version	769,558***	87	0.753	0.108	0.856	835.558
H1:15-3 modified original version	311,054***	66	0.914	0.074	0.940	419.054
H2:15-3PT	674,682***	87	0.787	0.100	0.879	740.682
**H2:15-3PT modified**	262,379***	**68**	**0.931**	**0.065**	**0.951**	**366.379**

*N* = 676. The model with the best fit is shown in bold. ***p < 0.001.

As shown in Table 3, the model “H2:15-3PT,” had a poor fit (*χ*^2^/d*f* [674,682/87] = 7.75; RMSEA = 0.100; CFI = 0.787; GFI = 0.879; AIC = 740.682). To improve it, was eliminated six outlier observations and we examined the modification indices (greater than 11; *p* < 0.001, Marôco, 2021b), respecting the theoretical considerations. The measurement errors of items 1, 2, 3, 4, and 5 of the wavering dimension and items 9, 10, 12, 13, 14, and 15 belonging to the flexible thinking dimension were correlated. In addition, correlations were considered between errors of items saturated in different factors, because, theoretically, according to Kim (2019), there are correlations between the dimensions. Specifically: correlations between errors in items 4 (wavering) and 6 and 7 (active adaptation), 1 (wavering) and 6 (active adaptation), 3 (wavering) and 15 (flexible thinking), 7 (active adaptation), and 13 (flexible thinking) and finally 8 (active adaptation) and 12 (flexible thinking) were assumed. In this way, it was possible to obtain a good fit quality (H2:15-3PT modified: *χ*^2^/d*f* [262,379/68] = 3.86; RMSEA = 0.065; CFI = 0.931; GFI = 0.951; AIC = 366.739).

The “H2:15-3PT-modified” model (Table 3) presents a better fit compared to the other hypothetical models, including the model that supports the distribution of items considering the original version of the inventory (H1:15-3 original version).

Regarding the convergent validity, Table 4 shows the factor loadings, composite reliability, and average variance extracted.

**Table 4:** Convergent validity (factor loadings, CR, and AVE).

Construct	Items	Factor loadings	CR	AVE
Wavering	1	0.644	0.79	0.430
	2	0.712		
	3	0.671		
	4	0.61		
	5	0.639		
Active adaptation	6	0.508	0.60	0.336
	7	0.701		
	8	0.512		
Flexible thinking	9	0.627	0.77	0.333
	11	0.717		
	12	0.693		
	13	0.642		
	14	0.513		
	15	0.309		

*N* = 676. CR, composite reliability; AVE, average variance extracted.

As shown in Table 4, most of the factorial loadings presented values above 0.5, and only items 10 and 15 obtained lower values, both items of the Flexible Thinking dimension. The composite reliabilities of the three dimensions obtained satisfactory values, that is, >0.6 (Hair et al., 2018), with the highest value in Wavering (0.79). AVE estimates show less satisfactory indices of convergent validity in the three dimensions, Wavering had also the highest AVE value (0.43).

### Career flexibility, time perspective and academic variables

Table 1 presents the descriptive statistics of the three subscales of CFI (based on the structure of the CFA results for this Portuguese sample), the four subscales of TPI, as well as the correlations between subscales and the reliability coefficients.

Analysis of the correlations between the three dimensions of CFI (Table 1) revealed a positive and significant correlation between active adaptation and flexible thinking (*r* = 0.35, *p* < 0.01), by contrast, wavering established low correlations both with flexible thinking and with active adaptation, suggesting the relative independence between these dimensions of CFI.

Reliability indices show that two dimensions of CFI presented adequate values of internal consistency, namely wavering (*α*, *ω* = 0.78) and flexible thinking (*α*, *ω* = 0.76), however, the active adaptation dimension presented a lower value (*α* = 0.60, *ω* = 0.61).

The analysis of correlations between career flexibility and time perspective dimensions (Table 1) shows that future orientation had a moderate positive correlation with active adaptation (*r* = 0.33, *p* < 0.01) and a negative moderate correlation with wavering (*r* = −0.49, *p* < 0.01). The wavering subscale established a moderate and positive correlation with the negative future orientation (*r* = 0.52, *p* < 0.01).

To explore possible differences between academic grades and the scientific area of study on time perspective and career flexibility, a Multivariate Analysis of Variance (One-Way MANOVA) was performed.

According to the results of One-Way MANOVA, there is a significant difference in the academic grades of on time perspective variables and flexibility variables, Pillai’s Trace = 0.047, *F* (28, 5,160) = 2,18, *p* = 0.001, partial *ƞ*2 = 0.01, (*π* = 0.999). Given this, an ANOVA for each of the dependent variables was performed (Table 5).

**Table 5:** Means, standard deviations, and one-way ANOVA statistics for ‘academic grades’ on career flexibility and time perspective variables.

Variable	E (*N* = 33)		D (*N* = 304)		C (*N* = 573)		B (*N* = 329)		A (*N* = 59)		F (4, 1,293)	*ƞ*2
	M	SD	M	SD	M	SD	M	SD	M	SD		
Career flexibility												
Wavering	13.64	3.55	12.79	3.93	12.18	4.04	11.59	3.85	12.41	4.63	4.73***	0.01
Flexible thinking	25.76	4.94	25.48	4.13	25.80	4.67	25.55	4.57	25.47	4.87	0.314	0.00
Active adaptation	9.39	2.56	9.14	2.14	9.29	2.12	9.57	2.17	9.73	1.95	2.12	0.01
Time perspective												
Future orientation	22.42	7.18	23.86	8.20	23.99	8.01	26.42	8.04	26.85	8.53	7.36***	0.02
Present orientation	25.42	5.65	23.27	6.97	23.47	7.13	22.33	7.34	21.14	7.29	3.39**	0.01
Past orientation	14.15	4.79	12.63	4.02	12.88	3.99	12.74	4.30	13.05	4.44	1.12	0.00
Negative future	14.45	6.01	12.01	6.20	11.30	5.79	10.35	5.51	10.54	5.33	6.02***	0.02

*N* = 1.298. M, mean; SD, standard deviation. E = values between 10–11. D = values between 12–13. C = values between 14–15. B = values between 16–17. A = values between 18–20. **p < 0.05. ***p < 0.001.

The descriptive statistics in Table 5 show that among the dimensions of career flexibility, wavering was the dimension with the greatest variability across the five grade levels, while among time perspective dimensions the variability among groups seems larger. ANOVA results (Table 5) indicate that, in fact, there are, statistically significant differences between ‘academic grades’ in one of the career flexibility variables, wavering (*F* (4, 1,293) = 4.73, *p* = 0.001, partial *ƞ*2 =. 01) and in three variables of the time perspective, future orientation (*F* (4, 1,293) = 7.36, *p* = 0.000, partial *ƞ*2 = 0.02), present orientation (*F* (4, 1,293) = 3.39, *p* = 0.01, partial *ƞ*2 = 0.01) and negative future (*F* (4, 1,293) = 6.02, *p* = 0.000, partial *ƞ*2 = 0.02).

The results of Tukey’s post-hoc HSD test indicated significant differences in wavering between the groups of grade level E (*M* = 13.64; DP = 3.55) and level B (*M* = 11.59; DP = 3.85); in future orientation between level A (*M* = 26.85; SD = 8.53), B (*M* = 26.42; SD = 8.04) and level E students with the lowest average score (*M* = 22.42; DP = 7.18). In negative future between level E (*M* = 14.45; SD = 6.01), and B grade level (*M* = 10.35; SD = 5.51. In the dimension of present orientation, the differences were significant between grade level E (*M* = 25.42; DP = 5.65) and A and B grade level students (A: *M* = 21.14; SD = 7.29, B: *M* = 22.33; SD = 7.34).

According to the results of One-Way MANOVA, there is also a significant difference of the factor scientific area of study on time perspective and career flexibility variables, Pillai’s Trace = 0.073, *F* (42, 8,232) = 2,41 *p* = 0.001, partial *ƞ*2 = 0.012, (*π*) = 1.000. Then, an ANOVA was performed for each of the dependent variables.

ANOVAs results (Table 6), indicated a significant difference of the ‘scientific area of study’ on two variables of career flexibility, wavering (*F* (6, 1,373) = 4.34, *p* = 0.001, partial *ƞ*2 = 0.02) and flexible thinking (*F* (6, 1,373) = 2.99, *p* = 0.01, partial *ƞ*2 = 0.01), and two variables from the time perspective, future orientation (*F* (6, 1,373) = 2.91, *p* = 0.01, partial *ƞ*2 = 0.01) and negative future (*F* (6, 1,373) = 6.99, *p* = 0.01, partial *ƞ*2 = 0.03).

**Table 6:** Means, standard deviations, and one-way ANOVA statistics for ‘scientific area of study’ on career flexibility and time perspective variables.

Variable	AS (*N* = 64)		EST (*N* = 294)		ES (*N* = 28)		MHS (*N* = 156)		NS (*N* = 54)		SS (*N* = 618)		Hu (*N* = 166)		F (6, 1,373)	*ƞ*2
	M	SD	M	SD	M	SD	M	SD	M	SD	M	SD	M	SD		
Career flexibility																
Wavering	11.44	3.63	12.85	4.13	14.71	4.27	11.62	4.01	11.98	3.36	12.05	3.93	12.04	4.05	4.34***	0.02
Flexible thinking	24.98	4.66	26.34	4.43	25.93	4.28	24.76	4.57	24.72	4.86	25.81	4.47	25.33	4.98	2.99**	0.01
Active adaptation	9.28	2.26	9.45	2.09	9.18	2.06	9.55	2.22	8.81	2.00	9.32	2.21	9.29	2.10	0.95	0.00
Time perspective																
Future orientation	24.63	7.88	23.67	7.59	21.25	7.65	26.27	8.49	24.48	8.38	25.05	8.13	24.18	8.77	2.91**	0.01
Present orientation	23.88	6.61	23.04	6.98	23.64	6.65	23.09	6.78	24.19	7.07	22.71	7.38	23.80	7.49	0.93	0.00
Past orientation	13.03	4.17	12.72	3.87	12.96	5.36	13.10	3.88	12.39	4.49	12.76	4.13	12.80	4.26	0.29	0.00
Negative future	12.50	5.81	11.80	5.71	14.89	6.03	10.24	5.69	11.33	6.55	10.64	5.67	12.95	6.38	6.99**	0.03

*N* = 1.380. M, mean; SD, standard deviation; AS, agricultural sciences; EST, Engineering Sciences and Technology; ES, exact sciences; MHS, Medical and Health Sciences; NS, Natural Sciences; SS, social sciences; Hu, Humanities. **p < 0.05. ***p < 0.001.

Tukey’s post-hoc HSD test shows statistically significant differences between the scientific areas in future orientation, namely between Medical and Health Sciences (*M* = 26.27; DP = 8.49) and Exact Sciences (*M* = 21.25; DP = 7.65). Significant differences in negative future appear between the scientific area of Medical and Health Sciences (*M* = 10.24; DP = 5.69), Social Sciences (*M* = 10.64; DP = 5.67) and Exact Sciences (*M* = 14.89; DP = 6.03).

Regarding career flexibility, there were differences in the wavering dimension between Exact Sciences, which had the higher score on this dimension (*M* = 14.71; DP = 4.27) and Agricultural Sciences (*M* = 11.44; SD = 3.63) and Medical and Health Sciences (*M* = 11.62; SD = 4.01), that had the lowest scores on this dimension.

Finally, in flexible thinking, statistically significant differences appeared between the groups of the scientific areas of Medical and Health Sciences (*M* = 24.76; DP = 4.57) and Engineering and Technology Sciences (*M* = 26.34; DP = 4.43).

## Discussion

CFI was designed by Kim (2019) to evaluate career flexibility, one skill essential for recognizing, creating, and using chance events as a career opportunity (Mitchell et al., 1999). To analyze the psychometric properties of the Portuguese version of CFI, exploratory and confirmatory factor analysis were performed. Results of the parallel analysis within EFA indicated that career flexibility can be operationalized with three factors, as Kim (2019) suggested. The three-factor structure of the CFI in the Portuguese sample explains more than half of the total variance, and although this value may be considered modest, this solution was accepted as the factors extracted, maintain the theoretical meaning and the same structure as the original version. It is worth mentioning that there are some divergences regarding the minimum threshold of explained variance to be considered relevant, for instance, Marôco (2021a) claims that there are no clear criteria to establish that value, but in practical terms, a minimum value of half the total variance may be acceptable.

EFA grouped all the items of the wavering dimension of CFI into factor 1. However, items 9 and 10 were allocated to the flexible thinking dimension in the present study, whereas they were originally assigned to the active adaptation. While both the active adaptation and flexible thinking dimensions aim to assess the active nature of career flexibility, they are theoretically differentiated as representing the proactive and reactive aspects, respectively (Kim, 2019). The exploratory factor analysis in this study suggests that some items may not have been elaborated enough to capture this distinction, leading to their grouping into different factors than expected. Nonetheless, it is noteworthy that these items remained within dimensions that represent the active nature of flexibility.

Regarding this conceptual differentiation of the active nature of flexibility, it is worth mentioning the studies by Griffin and Hesketh (2003). The authors proposed a conceptual approach to behavioral adaptability in work contexts, which they deemed essential for individual and organizational career development. Based on the Minnesota work-adjustment theory (Dawis and Lofquist, 1984), they defined adaptive behaviors as proactive and reactive. Proactive behaviors involve taking actions that positively impact the changed environment, while reactive behaviors involve modifying either the environment or oneself to better adapt to the new context. The authors considered both types of behavior to be active in nature. While Kim (2019) appears to have adopted a similar approach in characterizing proactive and reactive aspects as representing the active nature of career flexibility, the author did not provide a detailed explanation of the defining characteristics of these behaviors. Nonetheless, despite these aspects, the results of EFA in this study support the passive and active nature of career flexibility. Data from the CFA shows that the model with the best fit confirms the results of the EFA, which supports the conceptual structure of the CFI.

The AVE results of CFI indicate unsatisfactory convergent validity indices. However, in exploratory studies with new instruments, some authors (e.g., Rubia, 2019; Cheung et al., 2023) suggest that lower values of AVE can still be acceptable if additional criteria, such as the reliability coefficients and factor loadings, are met. With respect to the reliability of the CFI, the dimensions of flexible thinking and wavering exhibited acceptable internal consistency. In contrast, the active adaptation dimension displayed a less satisfactory value, highlighting the need for further revisions and refinement of this instrument.

The first set of hypotheses explored the relationship between time perspective and career flexibility, and the results partially supported some of them. Specifically, hypothesis 1 (H1) was partially supported, as the results show a positive and moderate relationship between future time perspective and adaptive adaptation, as expected. This suggests that students who focus on the future also have a proactive attitude toward changes in their long-term career goals (Kim, 2019). However, contrary to expectations, future time perspective did not establish a significant relationship with flexible thinking. One possible explanation for this result is that individuals who strongly focus on the future may plan their goals and aspirations in a committed way (e.g., Luyckx et al., 2010; Janeiro et al., 2017; Bennett et al., 2021), which can make them less prone to changing projects in the face of unexpected events. In short, future-oriented individuals can be proactive but may not necessarily be reactive when it comes to changing career goals.

As for H2, results show that wavering was significantly correlated with all dimensions of time perspective, but in different directions. A moderate and negative correlation was found between wavering and future orientation, suggesting that students who are more undecided and uncertain have less focus on the future. On the other hand, wavering also had moderate positive correlations with present orientation and with a negative view of the future.

The second set of hypotheses explored the relationships between career flexibility, time perspective, and academic variables. H3 expected that students with higher grade levels would present higher levels of future orientation, active adaptation, and flexible thinking, but this was only partially supported. In fact, this trend was only confirmed for time perspective variables, as students with future orientation had higher grade levels compared to students with negative future orientation, corroborating previous studies (e.g., Maksimovic et al., 2020; Bennett et al., 2021; Boo et al., 2022). Although there were no significant differences between academic grades groups for flexible thinking and active adaptation, suggesting a relative independence between these types of variables, significant differences emerged between academic grade levels for wavering, showing that students who scored high on wavering had the lowest grade levels, what can suggest the possible detrimental effect of wavering on academic involvement.

Regarding the scientific area of study (H4), differences were found in two career flexibility variables, namely in wavering and flexible thinking, and in two time perspective variables, future orientation and negative future orientation. Interestingly, students from Medical and Health Sciences had the highest scores on future orientation and the lowest on wavering, suggesting that these students have more confidence in their future, feel less hesitation between career choices, and seem also less flexible to consider changing their future plans, these results seem in line with the ones reported by Bennett et al. (2021).

On the other hand, students in exact sciences had the highest scores on the wavering subscale and the lowest scores on future orientation, suggesting greater uncertainty about their career plans. These differences between students of the various scientific areas may reflect the differences in expectations about employment and career opportunities among students. In fact, the impact of social, economic, and educational transformations, and even unplanned events, such as the Covid-19 pandemic (e.g., Kramer and Kramer, 2020; Venkatachary et al., 2020), have differential effects in the diverse fields of work and may affect the confidence or anxiety students feel about their career choices.

### Theoretical and practical implications

By investigating the multidimensionality of career flexibility this study contributes to the theoretical and practical understanding of the nature of career flexibility and its role in career development. Even if in an indirect way, our findings highlight the need for vocational psychology to be aware of the unpredictability of professional trajectories, considering the ongoing social, economic, and educational transformations (Skrbiš and Laughland-Booÿ, 2019; Barbulescu et al., 2022). In this sense, developing skills that promote a positive and open attitude towards uncertainty can help higher education students manage career-related challenges and improve their psychological well-being (Kim et al., 2020; Gati and Kulcsár, 2021). These skills can be considered important personal resources that can help students navigate the unpredictable events of their careers and everyday life (Valickas et al., 2019).

Our findings also suggest that higher education institutions should be aware of the importance of these skills, promote their development and take actions to help their students feel more confident in their professional choices and committed to their plans. This includes considering events that may affect the educational and professional path and are attributed to chance, as highlighted in previous studies (e.g., Barbulescu et al., 2022; Kim et al., 2023).

### Limitations and directions for future research

There are several limitations to this study that should be acknowledged. A first limitation refers to the sample that cannot be considered representative of college students in Portugal, thus limiting the generalizability of the findings. Since the CFI is a relatively new measure, re-evaluating its factorial structure in different samples and contexts in Portugal could be worthwhile. Moreover, because this study is cross-sectional, it cannot establish causality between variables, future studies may include longitudinal research to expand the understanding of the nature of the relationships between these variables. Another set of limitations refers to the results of the study. In fact, results showed some weaknesses of the CFI, namely the modest variance explained by the three-factor structure, unsatisfactory results regarding convergent validity, and the less satisfactory reliability coefficient of the active adaptation dimension. These limitations underscore the importance of refining the measurement instrument and deepening the understanding of the nature of career flexibility. Therefore, future studies are needed to provide new information on the validation of the CFI. Subsequently, studies should explore, through structural equation modeling, the effects between these variables, as well as the potential mediation and moderation effects with other variables.

Furthermore, the relative scarcity of literature supporting the findings, especially since the CFI is based on relatively recent career studies, emphasizes the need for further research to expand the understanding of PHT.

## Conclusion

This study aimed to adapt a new career flexibility inventory to the Portuguese context. The results indicated that the Portuguese version of the CFI has an adequate three-factor structure with good reliability indices. Some limitations regarding psychometric validity show the importance of further research to improve the measure. However, the findings contribute to theoretically and operationally deepening the discussions about the multidimensionality of Career Flexibility when approaching it from the perspective of the PHT.

Overall, the relationships between career flexibility and time perspective seem in line with theoretical indicators, showing that future orientation is positively correlated with active adaptation, and negatively with wavering, suggesting that students with a future focus are also more prone to an active adaptation and experience fewer doubts about career choices.

The analysis of results also showed differences in career flexibility and time perspective among groups of students of different scientific areas, which might suggest that the impact of uncertainty and unstable events may be perceived differently in the different fields of study and work. Unplanned events and uncertain contexts are a constant in our society and can significantly impact higher education students’ goals and career paths. By examining the relationship between career flexibility and time perspective, this study provides theoretical and practical insights for career counselors to help clients make the most of unexpected events and opportunities in uncertain contexts.

## Data availability statement

The raw data supporting the conclusions of this article will be made available by the authors, without undue reservation.

## Ethics statement

The studies involving human participants were reviewed and approved by Comissão Especializada de Ética e Deontologia do Conselho Científico da Faculdade de Psicologia. The patients/participants provided their written informed consent to participate in this study.

## Author contributions

JF and IJ contributed to the conception and design of the study and performed the statistical analysis. JF organized the database and wrote the manuscript sections. IJ contributed to the revision and writing the manuscript, read and approved the submitted version. All authors contributed to the article and approved the submitted version.

## Funding

This work received national funding from FCT – Fundação para a Ciência e a Tecnologia, I.P, through the Research Center for Psychological Science of the Faculty of Psychology, University of Lisbon (UIDB/04527/2020 and UIDP/04527/2020). This work also received national funding from FCT – Fundação para a Ciência e a Tecnologia through a Ph.D. grant awarded to the first author (2021.04614.BD).

## Conflict of interest

The authors declare that the research was conducted in the absence of any commercial or financial relationships that could be construed as a potential conflict of interest.

## Publisher’s note

All claims expressed in this article are solely those of the authors and do not necessarily represent those of their affiliated organizations, or those of the publisher, the editors and the reviewers. Any product that may be evaluated in this article, or claim that may be made by its manufacturer, is not guaranteed or endorsed by the publisher.
